# Biomechanical Strategies Underlying the Robust Body Armour of an Aposematic Weevil

**DOI:** 10.3389/fphys.2018.01410

**Published:** 2018-10-09

**Authors:** Lu-Yi Wang, Hamed Rajabi, Nima Ghoroubi, Chung-Ping Lin, Stanislav N. Gorb

**Affiliations:** ^1^Functional Morphology and Biomechanics, Institute of Zoology, Kiel University, Kiel, Germany; ^2^Department of Life Science, National Taiwan Normal University, Taipei, Taiwan; ^3^Young Researchers and Elite Club, Rasht Branch, Islamic Azad University, Rasht, Iran; ^4^Laboratory of Forest Ecology, Graduate School of Agriculture, Kyoto University, Kyoto, Japan

**Keywords:** aposematic color, cuticle microstructure, defense mechanism, material property, *Pachyrhynchus sarcitis kotoensis*, stiffness

## Abstract

Robust body armor is one of many anti-predator strategies used among animal taxa. The exoskeleton of insects can serve as the secondary defense mechanism in combination with the primary defense such as warning color. Aposematic *Pachyrhynchus* weevils advertise their unprofitability and use their robust exoskeleton for effective defense against lizard predators. While the mature weevils survive after the predatory attack, the soft teneral ones can easily be consumed. To reveal how the mature weevils achieve such effective protection, we investigated the ontogenetic changes in the microstructure and material properties of the exoskeleton of the adult weevils. We also tested the functional role of a weevil-specific structure, the fibrous ridge, in the robustness of the elytral cuticle of the mature weevils. The results showed that the mature weevils have thicker, stiffer and more sclerotized cuticle than the teneral ones. The fibrous ridges in the endocuticle considerably increase the overall stiffness of their cuticle. Together these biomechanical strategies enable *Pachyrhynchus* weevils to achieve robust body armor that efficiently protects them from lizard predation.

## Introduction

Well-defended aposematic prey animals advertise their unprofitability to potential predators by visual, acoustic or olfactory cues (Poulton, 1890; Ruxton et al., 2004). These cues often function as reliable warning signals through the linkage between the conspicuousness and an effective secondary defense. The defense strategies of aposematic preys can be divided into three categories based on the form of defense: chemical, behavioral, and morphological defense (Ruxton et al., 2004). Chemical defense is the most commonly known mechanism, in which aposematic animals possess toxic chemicals or repellents in their bodies or/and release them to predators when irritated. Behavioral defense is achieved by performing anti-predatory actions when threatened (e.g., starling display) (Ruxton et al., 2004; Stevens, 2013). For morphological defense, prey animals use mechanical protections, for example, spines or hard armor, to render themselves unpalatable (Ruxton et al., 2004; Stevens, 2013).

The Southeast Asian weevil genus, *Pachyrhynchus* (Germar, 1824) (Coleoptera: Curculionidae) is one of the best-known examples displaying colorful visual cue as a warning signal to advertise its robust body armor as a secondary defense against the predator lizards, *Japalura swinhonis* (Wallace, 1895; Schultze, 1923; Welch et al., 2007; Tseng et al., 2014; Wang et al., 2018). The flightless *Pachyrhynchus* weevils have a dome-shaped body armor, which is formed by firmly interlocked elytra. The force required to break the weevil's body was found to be significantly higher than the mean bite forces of the local lizards (Wang et al., 2018). *Pachyrhychus* weevils leave their pupal chambers when their bodies are still soft (teneral stage), so that teneral weevils can easily be penetrated by lizard predators (Wang et al., 2018). It takes at least 4 weeks for the teneral weevils to be fully hardened (Wang et al., personal observation). During this time, they are protected from experienced predators mainly by their primary defense of asposematic coloration.

The insect cuticle becomes gradually hardened after the eclosion. The deposition of endocuticle (Neville, 1963), melanization (Andersen, 2012), and dehydration of cuticle (Enders et al., 2004; Klocke and Schmitz, 2011; Dirks and Taylor, 2012) are likely to be the main reasons for the differences of cuticle stiffness. Aside from sclerotization (Hopkins and Kramer, 1992), the component of the cuticle (e.g., heavy metals; Schofield et al., 2002) and the geometry of the whole organism (Rajabi et al., 2014) could also potentially increase the mechanical strength of the structure. Previous studies have tracked the developmental changes in the biomechanical properties of cuticle in insects, such as grasshoppers (Neville, 1963), locusts (Hepburn and Joffe, 1974; Parle and Taylor, 2017), stick insects (Schmitt et al., 2018), and honeybees (Thompson and Hepburn, 1978). However, a comprehensive ontogenetic study on the structural and material strategies to achieve the exceptionally robust exoskeleton in the small-sized *Pachyrhynchus* weevils remains elusive.

This study analyzed the differences in biomechanics of the body armor between the mature and teneral *P. sarcitis kotoensis* for their effects under the mechanical impact of predator attacks. We quantified the difference in load carrying capacity and stiffness between the mature and teneral *P. sarcitis kotoensis*, and compared the microstructure and material composition of their body armor. By comparing the stiffness of the weevil cuticle with that of the teeth of the lizard predators and their bite force, we also aimed to answer whether it is the limitation from the dental property or the organismal performance that prevents the lizards from breaking the exoskeleton of the mature *Pachyrhynchus* weevils.

## Materials and methods

### Ethics statement

The permission to use the protected *P. sarcitis kotoensis* was granted by the Forestry Bureau, Council of Agriculture, Taiwan (no. 1060241435). The experiments comply with the ethical guidelines at National Taiwan Normal University (no. 105012) and Kiel University. The experiments were performed following the Ordinance on Safety and Health Protection at Workplaces Involving Biological Agents (BioStoffV) launched by Federal Ministry of Labor and Social Affairs, Germany.

### Sample preparation

Three pairs of adult *P. sarcitis kotoensis* were collected from Orchid Island, Taiwan in March of 2017 and reared individually in plastic containers (8 cm diameter, 6 cm height) with weekly supply of fresh leaves of *Leea guineensis* (Leeaceae) in the laboratory of National Taiwan Normal University at 25°C and under 12:12 h light:dark cycles. The eggs and the first instar larvae were reared inside the stems of *L*. *guineensis*. The “teneral” adult weevils were defined as the individuals still within 5 days of emergence from the pupal chambers; the “mature” adult weevils were defined as those having been emerged from the pupal chambers for more than 2 months (Wang et al., 2018). All weevil samples for the analyses were frozen at −20°C, except for those of cryo-SEM which were frozen at −70°C before the treatment. The cuticle of the weevil was removed using razor blades for nanoindentation test and CLSM scanning. The lizard specimen of *J. swinhonis* was caught in Mt. Dadu, Taichung, Taiwan in October of 2015 and frozen at −20 °C until the preparation. The head of the lizard was removed and its muscle was dissected with blades. The head skeleton was cleaned with hydrogen peroxide and kept at the room temperature until the analysis.

### Compression testing

**Figure 1:**
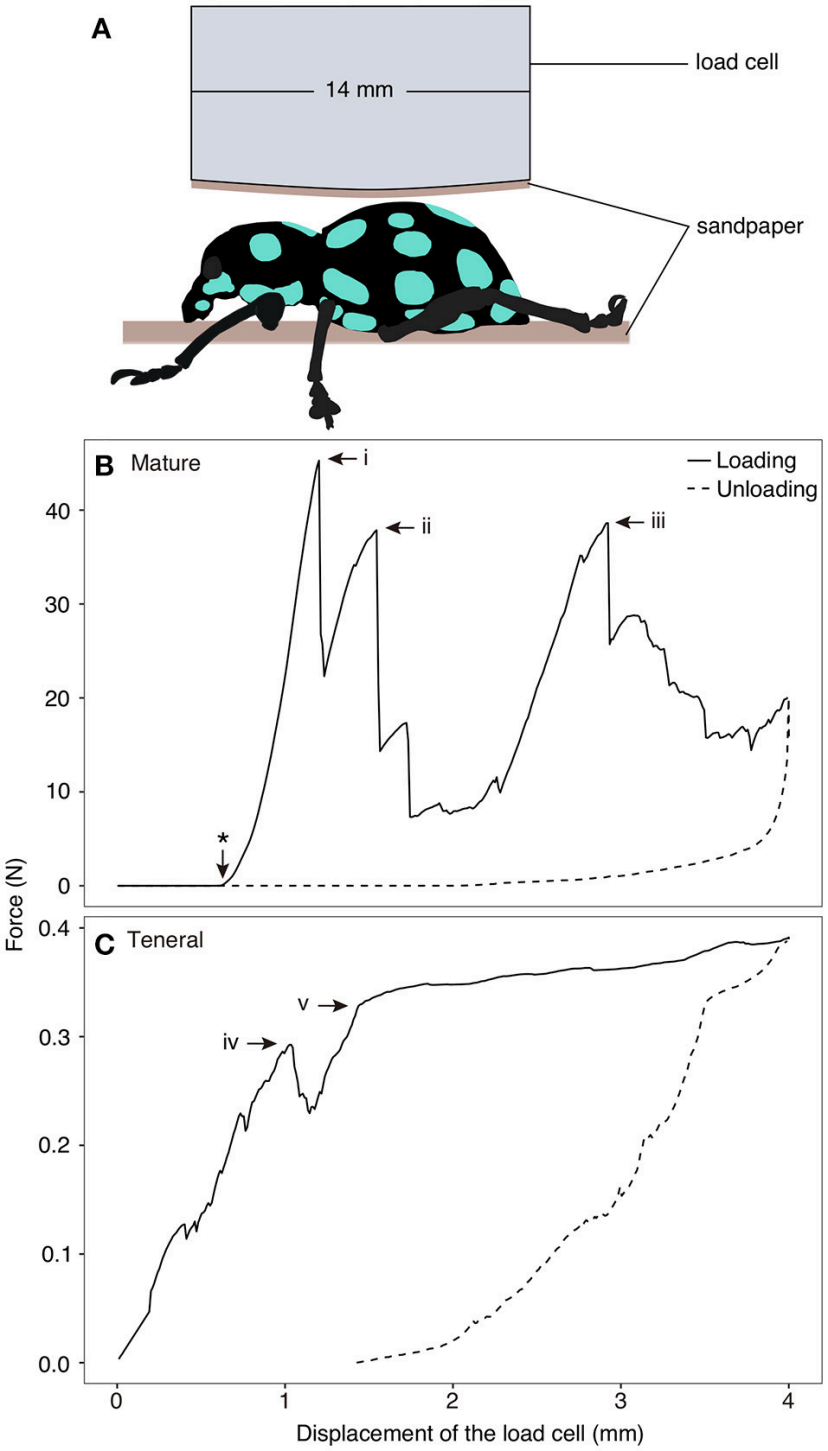
Compression test on *Pachyrhynchus sarcitis kotoensis*. **(A)** Weevil specimens were placed under the load cell. A piece of sandpaper was glued at the tip of the load cell and another piece was placed below the specimen to prevent its slippage. **(B)** Representative force-displacement curve obtained from compression test on a mature weevil. Load cell came in contact with the specimen after 0.62 mm displacement (marked by ^*^ on the curve). The force-displacement curve of the mature weevil has three force peaks (i: *F* = 45.27 N; ii: *F* = 37.85 N, iii: *F* = 38.61 N). Each drop in the force represents sudden crack propagation in the exoskeleton of the weevil (Movie S1). **(C)** Representative force-displacement curve obtained from compression test on a teneral weevil. The first crack in the exoskeleton occurred at iv (*F* = 0.29 N) and the slope of the curve decreased after v (*F* = 0.39 N) (Movie S2).

Compression tests were performed using a ZwickiLine uniaxial compression testing machine (Zwick Roell, Ulm, Germany) equipped with a 500 N load cell (Xforce P load cell) on two mature and two teneral adults to measure the load-carrying capacity of the exoskeleton. After specimens were completely thawed at room temperature, they were placed in the center of a sample holder (102.34 × 89.79 mm). Two small pieces of rough sandpaper were glued at the surface of the sample holder and at the tip of the load cell (diameter: 14 mm) to avoid slippage of the specimens (Figure 1A). The load cell compressed the specimens dorsally at a constantly increasing displacement of 1 mm/min. After reaching the maximal displacement of 4 mm followed by a three-second pause, the displacement was constantly decreased at a speed of 5 mm/min until complete unloading was achieved. The amount of the force and displacement were recorded using the test software testXpert (v. 3.5, Zwick Roell, Ulm, Germany). The testing process was recorded using a camera (D5300, Nikon Co., Tokyo, Japan) equipped with a macro lens (EF100 mm, Canon Inc., Tokyo, Japan).

### Cryo-scanning electron microscopy(Cryo-SEM)

The elytral samples of the weevils were separated from their bodies (one mature and one teneral individual). The specimens were broken into smaller pieces at −140°C in the cryo-preparation prechamber of a Hitachi S-4800 SEM (Hitachi High-Tech., Tokyo, Japan), and then coated with gold-palladium (thickness: 9 nm) using a Gatan ALTO 2500 cryo-preparation system (Gatan, Abingdon, UK). The coated samples were observed in the SEM at the temperature of −120°C and an accelerating voltage of 3 kV. The measurements of the cuticle microstructure were taken using the SEM images in ImageJ (https://imagej.nih.gov/ij/).

### Finite element modeling

**Figure 2:**
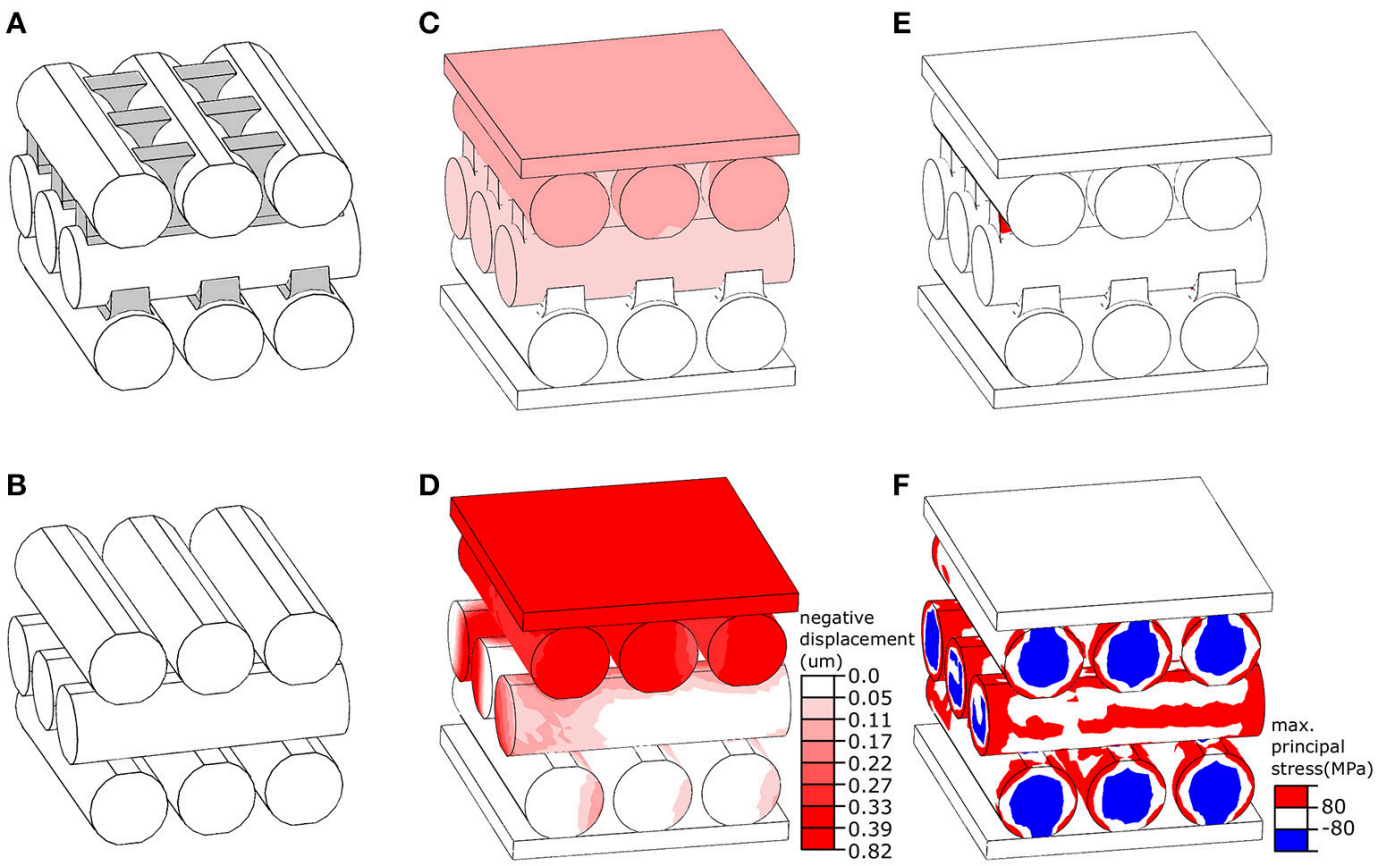
Numerical simulation of the mechanical behavior of layers in the elytral endocuticle of *Pachyrhynchus sarcitis kotoensis*. **(A)** The “*reference model*” represents a fraction of the endocuticle. The space between the microfibers in the upper layer is filled with ridges extended from microfibers in the lower layer. The ridges are shown in gray. **(B)** The “*no ridge model*” was developed by the removal of the ridges from the “*reference model*.” **(C,D)** Displacement of the “*reference model*” model **(C)** and “*no ridge model*” **(D)** under the same loading condition. The “*no ridge model”* experiences a displacement of about five times larger than that of the “*reference model*.” **(E,F)** Probability of damage (predicted by stresses higher than the strength of cuticle material) in the “*reference model*” **(E)** and “*no ridge model*” **(F)**. The presence of ridges substantially reduced the risk of damage in the microfibers.

The finite element (FE) software package ABAQUS/Standard v6.14 (Simulia, Providence, RI, USA) was used to develop a numerical model of the elytral cuticle based on the results of cryo-scanning electron microscopy. The model consisted of three layers, each having three parallel microfibers (Figure 2A). The microfibers of the center layer were positioned perpendicular to those of the top and bottom layers, providing a simplified representation of the orientation of microfibers in the endocuticle. All microfibers in the model had the same diameter (4 μm) and length (14 μm). The gaps between the microfibers in the upper layer were partly filled with ridges (Figure 2A, marked in gray) emerging from microfibers of the layer below. We refer to this model as “*reference model”* (Figure 2A). A second model, called “*no ridge model”* (Figure 2B) was developed by removing the ridges from the “*reference model*.” The OBJ files of the models are available as supplementary material (Datasheets 1, 2).

The models were placed between two rigid plates, as shown in Figures 2C,D. While the plate at the bottom of the models was fixed in all directions, the plate located at the top was able to move only normal to their surface. The models were subjected to the same loading condition by applying a compressive point force of 4 mN to the center of the plate above. The microfibers in both models were assumed to have the same material properties as those of the sclerotized cuticle. Their Young's modulus, tensile/compressive strength, Poisson's ratio and density were taken as 7.5 GPa (Rajabi et al., 2017), 80 MPa (Dirks and Taylor, 2012), 0.3 (Combes and Daniel, 2003), and 1200 kg/m^3^ (Dirks and Taylor, 2012), respectively. The microfibers were meshed using the general purpose tetrahedral elements, C3D4, with second-order accuracy. To improve performance of these elements, they were used in a very small size, resulting in a total number of 10,000 elements in each microfiber. The sliding of the microfibers was prevented by defining a “rough” friction between their interacting surfaces (ABAQUS V6.7, 2007). A dynamic explicit analysis was used to simulate the displacement of the models and the stress distribution within them.

### Confocal laser scanning microscopy (CLSM)

Small pieces (~1 × 3 mm) of elytral samples were removed from one mature and one teneral weevil with sharp razor blades. The samples were washed with 75% ethanol, embedded in glycerol, placed on a slide glass and covered with a cover glass. The confocal laser scanning microscope (Zeiss LSM 700, Carl Zeiss Microscopy, Jena, Germany) with a 20 × objective lens (Zeiss Plan-Apochromat; numerical aperture = 0.8) was used to visualize the material composition of the samples based on their autofluorescence. The CLSM was equipped with four stable solid-state lasers (wavelength: 405, 488, 555, and 639 nm) and four corresponding emission filters (BP420–480, LP490, LP560, and LP640 nm). The emitted wavelengths of 420–480 nm (blue) represent resilin-dominated cuticle, those >490 nm (green) correspond to less sclerotized and more chitinous cuticle, and those with wavelengths >560 nm and >640 nm (red) indicate highly sclerotized cuticle (Michels and Gorb, 2012).

### Nanoindentation test

The Young's moduli of the cuticle of the teneral and mature weevils and that of the teeth of the lizard were measured by nanoindentation tests, which is commonly used to measure insect cuticle stiffness (Barbakadze et al., 2006; Sun et al., 2008; Dai and Yang, 2010). The pieces of elytral cuticle (~2 × 2 mm) were removed from five mature and five teneral weevil individuals with sharp razor blades (0.23 mm thick, single-edge blade; Personna, Verona, Virginia, USA) and then glued (ergo 5925, Kisling, Zurich, Switzerland) to the cylindrical sample holders (diameter: 12.74 mm; height: 12.55 mm) (Figures S1A,B). The lizard's jaw was cut into pieces (~3 × 5 mm; with 2 or 3 teeth on the same jaw piece) by an electrical saw. The jaw pieces with several teeth were glued from the lateral side to the sample holders (Figure S1C). In order to create a flat surface at the highest part of the sample, we glued several small pieces of cover glass partially beneath the jaw pieces to level the lateral part of the teeth surface horizontally. After the glue was completely dried (~10 min.), the samples were placed into the chamber of the nanoindenter (Nano Indenter SA2, MTS Nano Instruments, Oak Ridge, TN, USA) equipped with a standard Berkovich indenter tip. The samples were loaded under constant velocity of 30 nm/sec and we averaged the stiffness measured at the penetration depth from 1.0 to 1.3 μm and 0.8 to 1.0 μm for the elytral and tooth samples, respectively. The Young's moduli were obtained from the slope of the unloading part of force-displacement curves. One to three elytral pieces of each weevil were tested. On each elytral sample, six to sixteen locations were measured. Five teeth from the upper jaw and seven teeth from the lower jaws of the lizards were tested. Two to six locations were measured for each tooth sample (Table S1). The Young's moduli obtained from measurements on the same samples were averaged and the mean values were used in the statistical analyses.

### Statistical analyses

Welch's *t*-test was performed to compare the whole cuticle thickness between the mature and teneral weevils. Two-sample *t*-test was used to compare the angles of the orientation of the microfibers between successive layers in the mature and teneral cuticle. Welch's analysis of variance (Welch's ANOVA) was conducted to analyse the Young's moduli of the lizard tooth and that of the elytral cuticle of the mature and teneral weevils with subsequent Games-Howell *post-hoc* test for multiple comparisons between groups. All statistical analyses were carried out in R 3.4.3 (R Core Team, 2017).

## Results

### Force resistance of body armor

The force-displacement curves obtained from the compression tests revealed distinct signatures of force resistance between the mature and teneral weevils (Figures 1B,C). In Figure 1, one representative result is shown for both the mature and teneral weevils, since the two experimental trials were consistent for the same type of the weevils. Each drop in the curves represented rapid crack propagation in the exoskeleton of the weevils (Figures 1B, i–iii, C, iv). The compression on the mature weevil constituted of three relatively high force peaks in sequence (Figure 1B; Movie S1). The first peak appeared at the displacement of 0.68 mm with the maximum force of 45.27 N (Figure 1B, i) (the displacement here indicates that of the load cell between the position where it first physically contacted the specimen, and the position where it was, when the crack took place. The same definition of the displacement is the same in the following context). This is the compressive force needed to initiate fracture of the abdomen in a mature weevil. The second peak occurred at 0.93 mm with a force of 37.85 N (Figure 1B, ii). The third peak was at 2.30 mm with a force of 38.61 N (Figure 1B, iii).

For the teneral weevil, the force-displacement curve showed a linear increase in the force by increasing the applied displacement (Figure 1C; Movie S2). The first peak in the curve occurred at 1.04 mm with a force of 0.29 N (Figure 1C, iv). The second peak was observed at a displacement of 1.43 mm with a force of 0.32 N (Figure 1C, v). Although the slope of the force-displacement curve notably decreased at this point, the force still increased until a maximum displacement of 4 mm was reached. Overall, the results of the compression tests showed that the exoskeleton of the mature weevil had a much higher load-carrying capacity than that of the teneral one, when compressed dorsally. The force, at which the failure of the mature weevil initiated, was 156.1 times higher than that of the teneral one.

### Microstructure of the cuticle

**Figure 3:**
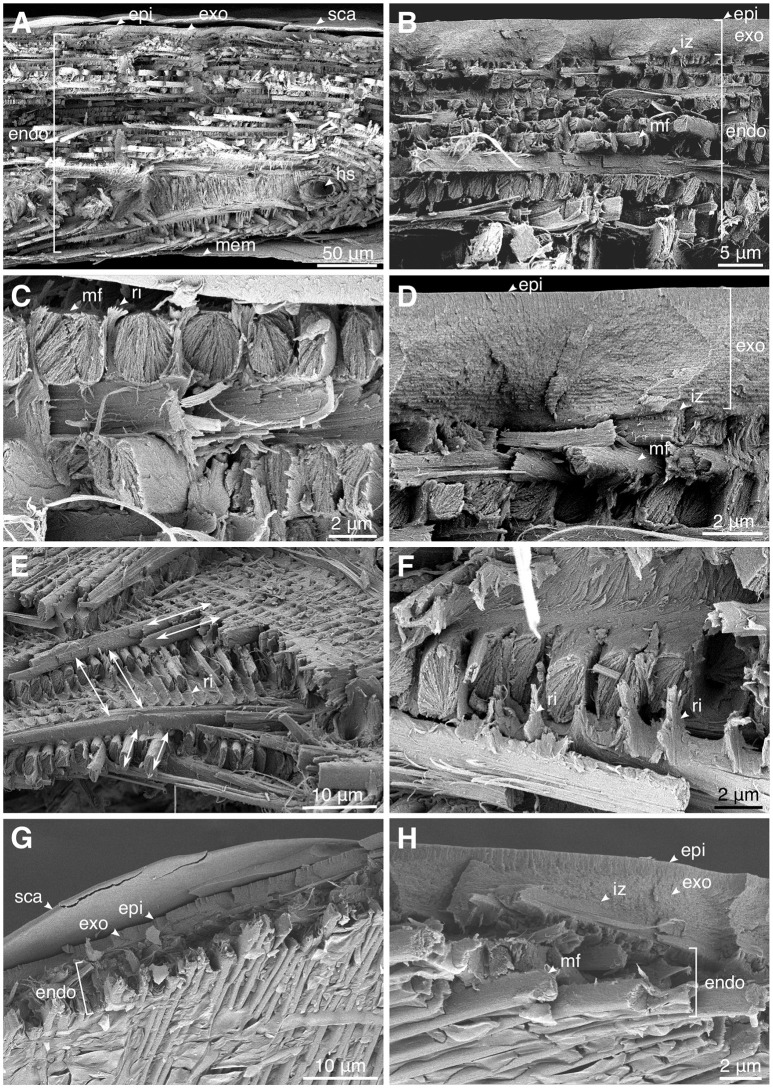
Microstructure of the cuticle of the mature **(A–F)** and teneral **(G,H)** *Pachyrhynchus sarcitis kotoensis* observed by cryo-scanning electron microscopy (cryo-SEM). **(A)** The cross section of the entire elytral cuticle of a mature weevil. **(B)** The size of the microfibers increases toward the middle of the endocuticle. **(C)** The microfibers interlocked by the ridges extending from the microfibers in the layer below. **(D)** The interlocking zone between exo- and endocuticle. **(E)** Angles between successive microfiber layers in the prothorax of the mature weevil. Arrows indicate the orientation of the microfibers. **(F)** The fibrous ridges extend perpendicularly from microfibers and surround microfibers in the upper layer. **(G)** The cross section of the entire elytral cuticle of a teneral weevil with only a few layers. **(H)** The interlocking zone between exo- and endocuticle. (epi, epicuticle; exo, exocuticle; endo, endocuticle; hc, hemolymph channels; mem, membrane; mf, microfiber; ri, ridge; iz, interlocking zone; sca, scale).

The elytral cuticle of *P. sarcitis kotoensis* could be divided into two parts based on its microstructure: (1) a highly dense layer of epi- and exocuticle and (2) a less dense layer of endocuticle with distinguishable microfibers. The epicuticle was so thin that it was difficult to identify its boundary with exocuticle (Figures 3D,H, epi & exo). There was also no clear border between exo- and endocuticle, so that these two layers seemed to be firmly bounded (Figures 3B,D,H, iz). Here, we used the term “*interlocking zone*” for the transition zone between exo- and endocuticle, adopted from Van de Kamp et al. (2016).

The mature weevil had a significantly thicker cuticle than the teneral one (entire cuticle, mature: 190.26 ± 4.22 μm; teneral: 8.22 ± 0.64 μm; *t* = 141.31, *p* < 0.001). The thickness of the epi- and exocuticle (together) and the thickness of the endocuticle in the mature weevil were 4.87 ± 0.56 μm and 172.74 ± 11.54 μm, respectively, while in the teneral weevil were 3.70 ± 0.44 μm and 6.92 ± 27 μm, respectively. Although the thickness of the epi- and exocuticle differed only slightly between the mature and teneral weevils, the endocuticle of the mature weevil was ~25 times thicker than that of the teneral one.

The exocuticle in both the mature and teneral *P. sarcitis kotoensis* was composed of many dense and thin sublayers, which seemed to have the typical helicoidal arrangement (Andersen, 1979). The endocuticle, on the other hand, was composed of packed layers of orderly lined oval microfibers (Figure 3C, mf), which were made up of numerous nanofibrils. The orientation of the microfibers within the same endocuticle layer was ~60° with respect to the successive layers (mature: 62.14 ± 13.40°; teneral: 54.53 ± 18.64°; *t* = 1.107, *p* = 0.280) (Figure 3E). The largest microfibers lied in the middle of the endocuticle and their sizes decreased toward the exterior and interior surfaces of the elytra (Figure 3A). Each microfiber was observed to have fibrous ridges perpendicularly extended toward the microfiber of the upper layer (Figures 3C,E,F). These ridges seemed to form a tight connection between the successive layers.

Aside from the thickness of the cuticle, the mature and teneral cuticle also differed in the number of layers and size of the microfibers. The endocuticle of the mature weevil had about 10 times more layers than that of the teneral one (mature: 36.00 ± 2.84 layers; teneral: 4.00 ± 0.00 layers) (Figures 3A,G). The average size of the microfibers of the mature weevil (width: 2.03 ± 0.51 μm, height: 3.13 ± 0.65 μm) was nearly 2 times larger than that of the teneral one (width: 1.02 ± 0.18 μm, height: 1.57 ± 0.61 μm). The larger microfibers found in the center of the endocuticle in the mature weevil had not yet been deposited in the teneral weevil.

### Numerical simulation

We used our developed FE models to investigate the mechanical function of ridges observed between the microfibers in the successive endocuticle layers (Figures 3C,E,F). Under a point force of 4 mN, the displacement of the “*reference model*” was less than 0.17 μm (Figure 2C) and resulted in a total strain of 1.42% (Figure 2E). The probability of damage of the “*reference model*” was minimal, and only a small region in the ridges experienced a stress exceeding the strength of the material (Figure 2E, red). Under the same loading condition, the displacement of the “*no ridge model*” equaled 0.82 μm (Figure 2D) and yielded a strain of 6.83% (Figure 2F), which was approximately five times larger than that of the “*reference model*.” A considerably higher level of stress than that of the “*reference model*” was observed in a wide area of this model (Figure 2F). In the most part of the model, the stress had exceeded the strength of the material (Figure 2F, red & blue). The maximal stress of the “*no ridge model*” (239 MPa) is about 3 times greater than that found in the “*reference model*” (80 MPa).

### Material composition and properties of the cuticle

**Figure 4:**
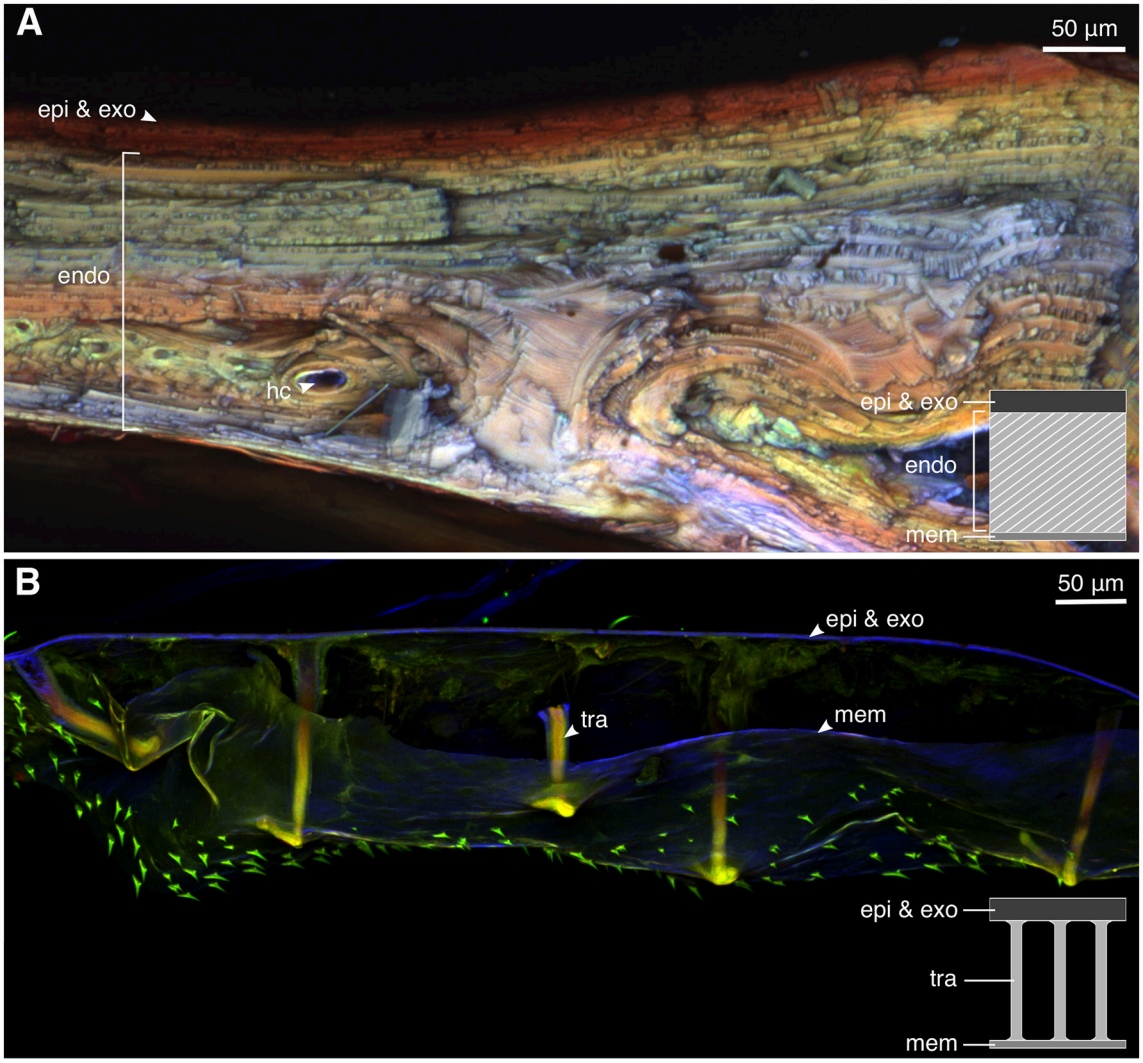
The material composition of the elytral cuticle of the mature and teneral *Pachyrhynchus sarcitis kotoensis* revealed by confocal laser scanning microscopy (CLSM). **(A)** Elytral cuticle of the mature weevil is thick and highly sclerotized (yellow, orange, and red). **(B)** Elytral cuticle of the teneral weevil is thin, resilin-dominated (blue), and contains sclerotized trabeculae (yellow) supporting the space between the cuticle and the inner membrane. (epi, epicuticle; exo, exocuticle; hc, hemolymph channels; mem, membrane; tra, trabecula).

The CLSM images indicated that the elytral exocuticle of the mature weevil is highly sclerotized in gerenal (Figure 4A, red). The exocuticle and a few exterior endocuticle layers showed a higher level of sclerotization than the layers below, indicated by their red autofluorescence. The interior layers of the endocuticle were less but yet sclerotized. This was evident from the green and yellow autofluorescence of these layers. Only a little blue autofluorescence could be found in the endocuticle, indicating low resilin content. The teneral weevil, in contrast, had a highly resilin-dominated cuticle (Figure 4B). As could be seen in Figure 4B, the whole cuticle thickness in the teneral individual was dominated by blue color. The trabeculae supporting the space between the cuticle and the inner membrane were the only regions showing a higher level of sclerotization (Figure 4B, tra).

**Figure 5:**
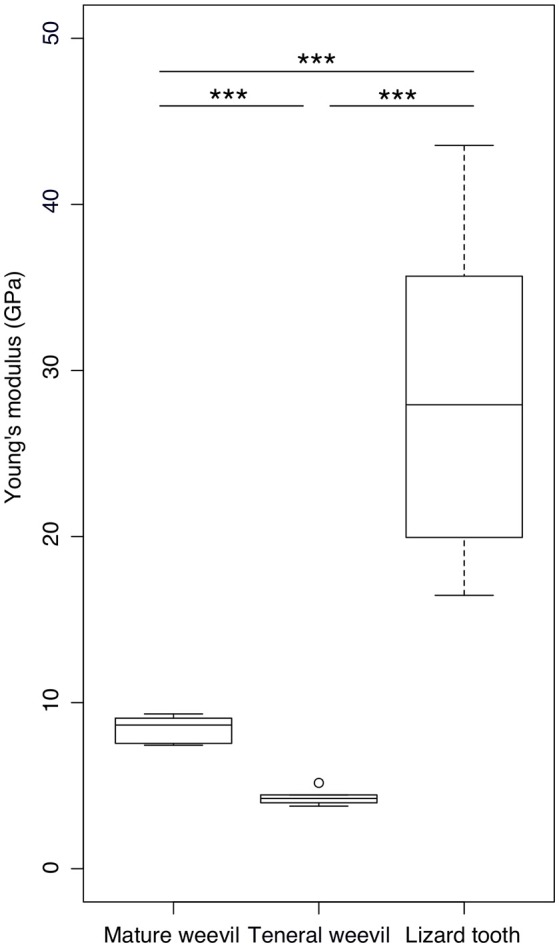
Comparison of the Young's modulus between the elytral cuticle of the mature and teneral *Pachyrhynchus sarcitis kotoensis* and the tooth of the lizard predator, *Japalura swinhonis*. The tooth of the lizard is significantly stiffer (28.81 ± 8.93 GPa) than the cuticle of both the mature (8.40 ± 0.87 GPa) and teneral weevils (4.32 ± 0.54 GPa). The mature cuticle is also significantly stiffer than the teneral cuticle (Games-Howell *post-hoc* tests, *p* < 0.001). The horizontal lines of the box show the upper whisker (the third quartile plus 1.5 times the interquartile range), the third quartile, the median, the first quartile, and the lower whisker (the first quartile minus 1.5 times the interquartile range) from top to down, respectively. The outlier is shown by a circle. Three asterisks indicate the significant level of *p* < 0.001.

According to the nanoindentation results, the mature cuticle having a Young's modulus of 8.40 ± 0.87 GPa (*n* = 5) was significantly stiffer than the teneral one with a Young's modulus of 4.32 ± 0.54 GPa (*n* = 5) (*t* = 8.952, *p* < 0.001) (Figure 5). The stiffness of the lizard tooth (the Young's modulus: 28.81 ± 8.93 GPa, *n* = 12) was significantly higher than both the mature (*t* = 7.823, *p* < 0.001) and teneral cuticle (*t* = 9.454, *p* < 0.001).

## Discussion

Both mature and teneral *Pachyrhynchus* weevils advertise aposematic coloration as primary defense against predation. However, when they encounter naïve lizard predators, the teneral weevils are easily consumed, while the mature weevils survive the attack without damage (Wang et al., 2018). The “secret weapon” of the mature weevils hides in their colorful exoskeleton. The elytral cuticle of the mature weevils is significantly stiffer than that of the teneral weevils. It requires ~150 times larger force to compress the exoskeleton of the mature weevils when compared with the teneral ones. Based on the results obtained from this study, we suggest that the weevils developed two key biomechanical strategies to achieve a stiff exoskeleton: structural and material strategies.

### Structural strategies

Although energetically costly, increasing the cuticle thickness seems to be a basic strategy used by the mature *P. sarcitis kotoensis* to enhance the total stiffness of their body armor. The cuticle of mature weevils is made up of a thick endocuticle containing numerous and densely packed microfibers (Figure 3A). This is different from the cuticle of teneral weevils, which was found to be significantly thinner and equipped with fewer and smaller microfibers in the endocuticle (Figures 3A,G).

The development of larger microfibers with oval shape cross sections is likely to be another adaptive strategy to increase the stiffness of the body armor in mature weevils. Comparing to the microfibers with circular cross sections, the oval microfibers have a larger second moment of area and, eventually, a higher flexural stiffness. On the other hand, the rotation of the microfibers in successive layers provides a homogeneous increase in the flexural stiffness in all directions of the abdomen rather than just one.

Van de Kamp et al. (2016) measured the diameters of microfibers in several weevil species. Among the flightless species they studied, the diameters of the oval microfibers ranged from 0.7 × 1.3 μm to 2.1 × 5.2 μm, which are comparable to the microfibers in the cuticle of *P. sarcitis kotoensis* (2.03 ± 0.51 μm × 3.13 ± 0.65 μm, mature weevils). In the mature *P. sarcitis kotoensis*, the microfibers in successive layers of endocuticle were bound together by a fibrous matrix forming protruding ridges, which interlock the successive layers (Figures 3C,F). The use of a set of numerical simulations enabled us to test the functional role of these ridges on the mechanical response of the endocuticle subjected to external loading. The results suggested that the fibrous ridges play an important role in both strengthening and stiffening the endocuticle. According to the FE models, although the ridges comprise merely ~6% of the total volume of the endocuticle, their removal resulted in 150% higher risk of structural damage and 80% reduction of the stiffness. This finding indicates that the presence of fibrous ridges is an effective biomechanical strategy to increase the mechanical resistance of the elytral cuticle.

### Material strategies

Although the degree of sclerotization varies across the thickness of the elytral cuticle of mature weevils, their cuticle is highly sclerotized in general. Specifically, one can find an unusually high degree of sclerotization in their endocuticle (Figure 4A; green, yellow, and orange). This is an interesting observation, especially when comparing with the results of previous studies indicating that the endocuticle in many insect species is soft and contains high proportion of resilin (termites: Varman, 1980; stick insects: Schmitt et al., 2018; dragonflies and damselflies: Appel et al., 2015; Rajabi et al., 2016). The increased sclerotization of the endocuticle, therefore, is likely to be an additional strategy to enhance the robustness of the body armor in *P. sarcitis kotoensis*.

The results of nanoindentation experiments confirmed the CLSM observations and indicated the significantly higher Young's modulus of the elytral cuticle of the mature weevils in comparison to that of the teneral ones. Particularly in the mature weevils, the maximum indentation depths achieved in the experiments were relatively small, if compared to the whole cuticle thickness (~0.52% in mature cuticle; ~10.84% in teneral cuticle). Therefore, we assume that the measured Young's moduli mainly reflect the properties of epi- and exocuticle layers, which are located near the surface of the cuticle. The comparison of the results of our measurements with similar nanoindentation data from the literature suggests that exterior layers in the elytral cuticle of *P. sarcitis kotoensis* have Young's moduli (8.40 ± 0.87 GPa) comparable with those of other beetles with relatively rigid cuticle (Table 1).

**Table 1:** The Young's moduli of beetles obtained by nanoindentation from the present study and the literature.

**Family**	**Species**	**Common name**	**Tested location**	**Young's modulus (GPa)**	**References**
Curculionidae	*Pachyrhynchus sarcitis kotoensis*	Weevil	Mature elytral cuticle	8.40 ± 0.87	Present study
			Teneral elytral cuticle	4.32 ± 0.54	
Scarabaeidae	*Pachnoda marginata*	Rose chafer	Gula (dry)	7.50 ± 1.80	Barbakadze et al., 2006
			Gula (wet)	1.5 ± 0.8	
	*Copris ochu*	Dung beetle	Foreleg tibia	3.74 ± 0.73	Sun et al., 2008
			Clypeus	15.98 ± 0.83	
			Elytra	7.24 ± 0.69	
			Prothorax	14.42 ± 0.44	
	*Holotrichia sichotana*		Foreleg tibia	3.97 ± 0.08	
			Clypeus	5.40 ± 0.26	
			Elytra	7.48 ± 0.69	
			Prothorax	12.56 ± 0.54	
	*Allomyrina dichotoma*	Japanese rhinoceros beetle	Elytra	8.16	Dai and Yang, 2010
	*Potosia brevitarsis*	White-spotted flower chafer beetle	Elytra	4.34	
Geotrupidae	*Geotrupes stercorarius*	Dung beetle	Foreleg tibia	7.61 ± 0.20	Sun et al., 2008
			clypeus	7.72 ± 0.26	
			Elytra	2.23 ± 0.36	
			Prothorax	8.89 ± 0.24	
Dytiscidae	*Cybister japonicus*	Diving beetle	Elytra	8.16	Dai and Yang, 2010
Lucanidae	*Serrognathus titanus*	Stag beetle	Elytra	5.42	

### Implications for lizard predation

The Young's modulus of the teeth of the lizard predator, *J. swinhonis*, was significantly greater than that of the elytral cuticle of both the mature and teneral *P. sarcitis kotoensis*. This suggests that the teeth as biting tools are capable of breaking the cuticle of the weevils without damage. Therefore, the failed predation of the lizard on the weevil cannot be explained by the stiffness of the lizard's teeth. This finding is also supported by earlier observations showing no damage to the lizard teeth after biting attempts on the weevil (Tseng et al., 2014; Wang et al., 2018). The force needed to initiate a crack on the exoskeleton of the mature weevil was 58.49 N, which is almost twice of the mean maximal bite force of lizard populations of *J. swinhonis* on the co-existing Green Island (males: 27.97 ± 9.55 N, *n* = 91; females: 8.14 ± 2.42 N, *n* = 80) and Orchid Island (males: 29.66 ± 9.62 N, *n* = 80; females: 12.03 ± 2.80 N, *n* = 89) (Wang et al., 2018). This explains why *J. swinhonis* lizard avoids feeding on the *Pachyrhynchus* weevils. Only very large lizards (~3% of the trials) are able to generate enough biting force to break the exoskeleton of the mature weevils (Tseng et al., 2014).

In this study, we examined microstructure and material properties of the cuticle in mature and teneral weevils. The results revealed the biomechanical mechanisms underlying the formation of the robust exoskeleton of the mature weevils. Not only the thick, stiff, and highly sclerotized cuticle, but also the presence of large microfibers and fibrous ridges interlocking the successive layers in the endocuticle are likely to play key functional roles in the robustness of the elytral cuticle. The combination of these factors enables the *Pachyrhynchus* weevils to achieve a robust body armor that efficiently protects them from predation by lizards. Since teneral *Pachyrhynchus* weevils have a relatively long soft stage after leaving pupal chambers, future research on the adaptation of teneral weevils having low degree of cuticle sclerotization is expected to bring further insights into the evolution of aposematism.

## Data accessibility

All data from this study are available from Figshare: https://figshare.com/s/62e16e8176ca86e3f6f6.

## Author contributions

L-YW participated in the conception of the study, design of the study, data collection, data analyses, and drafting the manuscript; HR participated in the design of the study, data collection, data analyses, revising the manuscript, and supervision; NG carried out the FE modeling; SG participated in the design of the study, taking SEM images, revising the manuscript, and supervision; C-PL participated in the conception of the study and revising the manuscript. All authors gave final approval for publication.

### Conflict of interest statement

The authors declare that the research was conducted in the absence of any commercial or financial relationships that could be construed as a potential conflict of interest.

## Supplementary material

Figure S1 The sketch of the samples for nanoindentation. **(A)** The mature cuticle. **(B)** The teneral cuticle. **(C)** The lizard tooth.

Table S1 The stiffness of the elytral cuticle of the mature and teneral weevil, *Pachyrhynchus sarcitis kotoensis*, and the tooth of the lizard, *Japalura swinhonis*.

Movie S1 Compression test on a mature *Pachyrhynchus sarcitis kotoensis*.

Movie S2 Compression test on a teneral *Pachyrhynchus sarcitis kotoensis*.

Datasheet 1 “Reference model.”

Datasheet 2 “No ridge model.”
