# Supplementary figures and images for: Biomechanical Strategies Underlying the Robust Body Armour of an Aposematic Weevil
Source: Front Physiol. 2018 Oct 9;9:1410. doi: 10.3389/fphys.2018.01410 (PMC6189447; doi:10.3389/fphys.2018.01410)

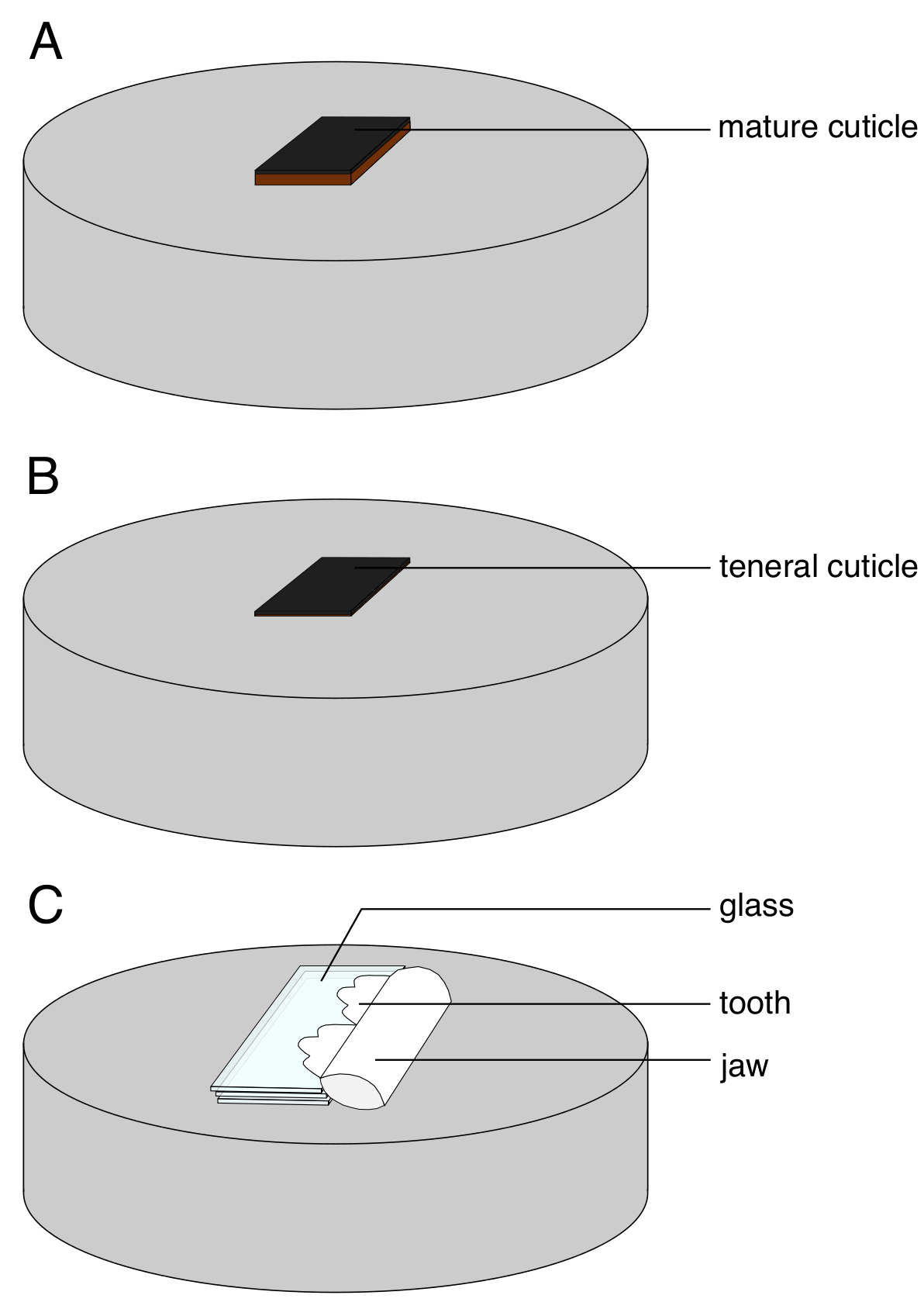

Supplement: Figure S1 — The sketch of the samples for nanoindentation. (A) The mature cuticle. (B) The teneral cuticle. (C) The lizard tooth. [file Image_1.TIF]
